# Impact of Olive Extract Addition on Corn Starch-Based Active Edible Films Properties for Food Packaging Applications

**DOI:** 10.3390/foods9091339

**Published:** 2020-09-22

**Authors:** Arantzazu Valdés García, Olga B. Álvarez-Pérez, Romeo Rojas, Cristobal N. Aguilar, María Carmen Garrigós

**Affiliations:** 1 Analytical Chemistry, Nutrition & Food Sciences Department, University of Alicante, P.O. Box 99, 03080 Alicante, Spain; mc.garrigos@ua.es; 2 Food Research Department, School of Chemistry, Universidad Autónoma de Coahuila, Saltillo 25280, Coahuila, Mexico; berenice.alvarez.perez@uadec.edu.mx (O.B.Á.-P.); cristobal.aguilar@uadec.edu.mx (C.N.A.); 3 Research Center and Development for Food Industries, School of Agronomy, Universidad Autónoma de Nuevo León, General Escobedo 66050, Nuevo León, Mexico; romeo.rojasmln@uanl.edu.mx

**Keywords:** antimicrobial activity, antioxidant activity, edible films, food packaging, olive extract, starch

## Abstract

Active edible films based on corn starch containing glycerol as a plasticizer and an olive extract obtained from Spanish olive fruit (*Olea europaea*) by-products (olive extract; OE) at different concentrations (0, 0.05, 0.1 and 0.2 wt%) were prepared by using the casting technique and further solvent-evaporation. OE showed high total phenolic and flavonoids contents and antioxidant activity, which was evaluated by using three different methods: free radical scavenging assay by (1,1-dipheny l-2-picrylhydrazyl) DPPH, 2,2-azino-bis (3-ethylbenzothiazoline-6-sulfonic acid) ABTS radical inhibition and ferric reducing antioxidant power (FRAP). The incorporation of OE into the corn starch/glycerol matrix underlined the antioxidant potential and antimicrobial effect against *E. coli* and *S. aureus* of these novel active films, being noticeable for films added with 0.2 wt% OE. The developed active films showed a clear thermo-oxidative stability improvement with OE incorporation, in particular at 0.2 wt% loading with an increase of around 50 °C in the initial degradation temperature (T_ini_) and oxidation onset temperature (OOT). The functional properties of control films were also improved with OE addition resulting in a decrease in Young’s modulus, elongation at break, shore D hardness and water vapor permeability. The present work suggested the potential of the developed corn starch-based edible films as low-price and sustainable food packaging systems to prevent the oxidative deterioration of packaged foodstuff while reducing also the generation of olive by-products.

## 1. Introduction

Bioplastics represent about 1% of the large amount (more than 359 million tons) of plastics produced worldwide. According to recent market data reported, bioplastics production was estimated to be around 2.11 million tons in 2019 with a forecast to increase to 2.43 million tons in 2024 [1]. In this context, bioplastics for packaging cover almost 70% (1.2 million tons) of the total bioplastics market, being up to 55.5% biodegradable plastics. It is also expected that their production will increase to 1.33 million tons in 2024. Nowadays, consumers are requiring natural, minimally-processed, healthy foods and ready-to-eat fresh products [2]. Thus, new strategies related to preservation techniques have been developed in recent years to protect food without hampering its organoleptic and nutritional properties. In this context, edible active films have increased in popularity since they can improve the quality, safety and functionality of food products [3].

Edible films are considered thin polymer layers and they are usually applied on food products to improve their preservation, distribution and marketing as environmentally friendly alternatives to synthetic films [4,5]. In particular, polysaccharides extracted from *Aloe Vera*, *Aloe Arborescens*, the mucilage of *Opuntia Ficus* and other sources have been used to produce edible biopolymer matrices with potential to extend food shelf-life [6,7,8,9]. Among them, starch is one of the most interesting biodegradable polysaccharides for food packaging because of its high availability, low cost and it being a natural origin of polymer materials with great ability to form transparent films without color and odor and its low oxygen permeability [10,11]. Starch comes from cereals such as corn and rice, legumes and tubers. Although its properties vary depending on the plant source [12], starch is basically composed of two polyglucans: amylopectin, which is the major component present in most starches with a branched structure consisting of short chains of α-(1, 4)-linked D-glucosyl units interconnected through α-(1, 6)-linkages, and the minor component (15–35 wt% of the total weight), amylose, a linear polymer formed by glucose units linked by α-(1, 4) bonds [13]. Nevertheless, starch has one main disadvantage since it shows high water vapor permeability due to its hydrophilic nature resulting from the presence of -OH groups in its structure, with a poor moisture barrier and mechanical properties, reducing its use for food packaging applications [14]. To overcome this drawback, lemongrass [15], cinnamon, clove [16,17] and orange [18] essential oils have been incorporated into starch matrices to reduce water vapor permeability. Among starches, corn, cassava and wheat have been mostly reported to be used in the formulation of biodegradable edible films due to their availability and relative low price [19,20,21].

The incorporation of natural additives coming from plants, essential oils or agricultural wastes into edible films is a trending topic in active materials research. Among them, materials based on the release of antioxidant and/or antimicrobial compounds obtained from natural sources are getting much interest in the food industry [22]. In this context, it has been reported that corn starch may serve as a carrier of antioxidant compounds present in zein–rutin composite nanoparticles [23], murta leaves [24], justiciar adhatoda leaf extract [25], and antimicrobials such as lauric arginate and natamycin [26], spice essential oils [27], cinnamaldehyde [28] and Aloe vera gel [29].

Olive is a widely consumed fruit due to its recognized nutritional value. The latest FAOSTAT data regarding the olive production in 2018 showed that around 21 million tons of olives were produced worldwide. Among them, Europe’s production was around 13 million tons, with Spain being the main producer followed by Italy, Greece and Portugal [30]. Previous studies have demonstrated the effectiveness of olive oil addition to reduce the water vapor permeability of chitosan-based biofilms [31]. Olive oil was also used as an active natural additive on sodium alginate [32] and chitosan-based biofilms [33,34], mainly due to the action of hydroxytyrosol and tyrosol compounds [35]. Regarding corn starch-based films, some studies have been recently reported in which compounds extracted from olive oil were selected as active additives [36]. However, no information has been found in the current literature concerning corn starch-based films incorporating an olive extract obtained from olive fruit (*Olea europaea*) by-products which usually contain pulp, oil, vegetative waters and bioactives. In this sense, oleuropein, one of the most abundant bioactive components present in the leaves and green fruits of the olive tree, is a phenolic secoiridoid glycoside that consists of a polyphenol, namely 4-(2-Hydroxyethyl) benzene-1, 2-diol, commonly known as hydroxytyrosol, a secoiridoid called elenolic acid and a glucose molecule that has been extensively reported for its antioxidant and antimicrobial properties, both in vitro and in vivo [37,38]. Then, the purpose of this work is the development of novel active edible films with antioxidant and antimicrobial activity based on corn starch with the addition of an olive extract (OE) from Spanish olive fruit by-products to be used for food packaging applications. OE was characterized in terms of total phenolic and flavonoid contents, and antioxidant, antimicrobial, structural and thermal properties. A complete physicochemical and functional characterization of the obtained active edible films added with different OE contents (0, 0.05, 0.1 and 0.2 wt%) was also performed. Finally, the study of the antioxidant and antimicrobial capacity of the developed active films for food packaging applications was evaluated.

## 2. Materials and Methods

### 2.1. Materials

Maizena^®^ was purchased from a local supermarket (Alicante, Spain) with 86 wt% carbohydrates, 1 wt% fiber, and less than 0.5 wt% lipids and proteins, without the presence of any preservatives or other additives. Glycerol (analytical grade) was obtained from Panreac (Barcelona, Spain). The olive extract (OE) was kindly provided by Probeltebio (Murcia, Spain) and it was obtained from Spanish olive fruit (*Olea europaea*) by-products after water extraction [39]. According to the supplier information, OE was mainly composed of 40.6 wt% hydroxytyrosol and it was used for the development of the active edible films.

Quercetin, gallic acid, aluminum chloride, potassium persulfate, sodium nitrite, sodium acetate trihydrate, Folin−Ciocalteu reagent (2 N), TPTZ (2,4,6-tripyridyl-s-triazine), DPPH (2,2-diphenyl-1-picrylhydrazyl) reagent and ABTS (2,2′-azino-bis (3-ethylbenzothiazoline-6-sulfonic acid)) diammonium salt were supplied by Sigma-Aldrich (Madrid, Spain). Ethanol, calcium chloride, iron trichloride, sodium hydroxide and sodium carbonate were supplied by Panreac (Barcelona, Spain). For the antimicrobial analyses, Gram-positive (*Staphylococcus aureus*, CECT 239) and Gram-negative (*Escherichia coli*, CECT 434) bacterial strains were purchased from the Spanish Type Culture Collection. Mueller–Hinton agar was obtained from BIOSER (Barcelona, Spain) and tryptic soy agar (TSA) was purchased from Sigma-Aldrich (Madrid, Spain).

### 2.2. Olive Extract Characterization

#### 2.2.1. Total Phenolic Content (TPC)

The total phenolic content (TPC), expressed as milligrams of gallic acid equivalents (GAEs) per gram of OE, was determined, in triplicate, by the Folin–Ciocalteu colorimetric method [40] using a Biomate-3 UV-Vis spectrophotometer (Thermospectronic, Mobile, AL, USA). In total, 50 µL of OE were mixed with 200 µL of Folin–Ciocalteu reagent. The mixture was vortexed and incubated for 5 min. Then, 1250 mL of a solution of 5 wt% Na_2_CO_3_ were added and mixed. Then, distilled water was added to bring the mixture to a final volume of 5000 µL. The absorbance was determined after 60 min of incubation in the dark at ambient temperature and 750 nm using deionized water as blank. Gallic acid was used as reference standard (25−1000 mg kg^−1^).

#### 2.2.2. Total Flavonoid Content (TFC)

The total flavonoid content was determined, in triplicate, [41] using the Biomate-3 UV-Vis spectrophotometer already described. A total of 30 µL of NaNO_2_ (10 wt%), 60 µL of AlCl_3_ 6H_2_O (20 wt%), 200 µL of NaOH (1 M) and 400 µL of distilled water were added to 100 mL of OE. The absorbance was measured at 510 nm using deionized water as blank. Quercetin was the reference standard (25−1000 mg kg^−1^) and the results were expressed as mg quercetin equivalents (CAE) per gram of OE.

#### 2.2.3. In Vitro Antioxidant Activity Assays

Antioxidants could act by several mechanisms and more than one method is needed to consider their different modes of action, e.g., by reducing power or free radical scavenging activity, donating hydrogen to radicals or metal chelating ability, among others [42]. Then, the antioxidant activity should not be evaluated from the results of a single antioxidant test [43] and, generally, different antioxidant tests using free radical traps are combined in vitro to obtain information about the antioxidant activity of the extracts. In this work, the antioxidant activity of OE was tested by three different methods: free radical scavenging assay by the (1,1-dipheny l-2-picrylhydrazyl) DPPH· method, the 2,2-azino-bis (3-Ethylbenzothiazoline-6-sulfonic acid) ABTS^+^ radical inhibition and the ferric reducing antioxidant power (FRAP) methods. All methods were performed in triplicate and the resulting solutions were tested by using a Biomate-3 UV-Vis spectrophotometer. Results were expressed as the percentage of inhibition (%) and they were calculated as described in Equation (1):

Inhibition (%) = [(Abs_control_ − Abs_sample_)/Abs_control_] × 100 (1)

The DPPH assay is a simple, low-time consuming and economic method used to evaluate the antioxidant activity that involves the transfer of hydrogen atoms. In this work, this test was carried out by following the methodology proposed by Molyneux [44]. In brief, 2900 µL of 60 µM DPPH· radicals freshly prepared were added to 100 µL of OE in assay tubes. The absorbance was measured after 30 min of incubation in the dark at ambient temperature at 517 nm using deionized water as blank.

The FRAP assay is based on the reduction caused by antioxidants to reduce the yellow complex Fe^3+^ into Fe^2+^ blue complex in the presence of TPTZ. The reaction is pH-dependent (with an optimum pH of 3.6, which was selected in this study). However, FRAP cannot detect species that act by hydrogen transfer, particularly thiols, glutathione and proteins [45]. Firstly, the FRAP reagent was prepared by a mixture of 0.1 mol L^−1^ sodium acetate buffer (pH 3.6), 10 mmol L^−1^ TPTZ and 20 mmol L^−1^ of ferric chloride (10:1:1). Then, 900 µL of the FRAP solution was added to 90 μL of distilled water and 30 µL of OE. Finally, the absorbance was measured immediately after the addition of the sample at 593 nm at 25 °C.

The ABTS assay is one of the most common methods to determine the antioxidant capacity of lipophilic and hydrophilic samples as the radical is soluble in organic solvents and water. A total of 12.5 mL of 2.45 mM potassium persulfate was mixed with 25 mL of a 7 mM ABTS solution to form the radical ABTS^+^. After 16 h of incubation in the dark at ambient temperature, the absorbance was measured at 734 nm [46]. To avoid obtaining saturated solutions, the ABTS^+^ solution was diluted with ethanol until reaching an initial absorbance value of 0.70 ± 0.01. Then, 950 µL of the ABTS^+^-adjusted solution was added to 50 µL of OE and the absorbance was immediately measured at 734 nm using distilled water as blank.

#### 2.2.4. Antimicrobial Activity

The antimicrobial activity of OE was investigated against Gram-positive (*Staphyloccocus aureus*, CECT 239) and Gram-negative (*Escherichia coli*, CECT 434) bacterial strains, by the agar-plate diffusion method in sterile Petri dishes. Suspensions of these microorganisms were spread onto Mueller–Hinton agar plates inoculated with two different concentrations of OE (1 and 5 wt%). After incubation at 37 °C for 24 h, the counting of the total CFU was carried out. Positive controls were used with a concentration of 10^6^ CFU mL^−1^ of each microorganism, following the methodology proposed by Ramirez et al. [47] and Janakat et al. [48].

#### 2.2.5. Attenuated Total Reflectance-Fourier Transform Infrared Spectroscopy (ATR-FTIR)

The ATR-FTIR spectra of OE (2 ± 0.01 mg) were registered, in triplicate, in the absorbance mode (4000–600 cm^−1^) using a Bruker Analitik IFS 66 FTIR spectrometer (Ettlingen, Germany) as it was described by Valdés et al. [49].

#### 2.2.6. Thermal Stability

The thermal stability of OE was studied, in triplicate, by thermogravimetric analysis (TGA) and differential scanning calorimetry (DSC). TGA tests were carried out using a TGA/SDTA 851 Mettler Toledo (Mettler Toledo, Schwarzenbach, Switzerland) thermal analyzer. A total of 5.0 ± 0.1 mg of OE was heated from 30 to 850 °C at 5 °C min^−1^ under nitrogen atmosphere (50 mL min^−1^). Two thermal parameters were determined: the initial degradation temperature, T_ini_ (°C), calculated at 5% of weight loss, and the temperature of maximum degradation, T_max_ (°C), corresponding to the maximum decomposition rate. Dried OE after 24 h at 60 °C was also analyzed by TGA.

DSC tests were conducted with a TA DSC Q-2000 instrument (TA Instruments, New Castle, DE, USA) under nitrogen atmosphere (50 mL min^−1^). In total, 8.0 ± 0.1 mg of OE was introduced in aluminum pans and they were cooled from 30 °C to −80 °C (3 min hold), heated to 250 °C (3 min hold), cooled to −80 °C (3 min hold) and finally heated to 250 °C (3 min hold), with all steps at 10 °C min^−1^ [50]. Calorimetric curves were analyzed in the second heating scan with the Universal Analysis TM Software (TA Instruments, New Castle, DE, USA) to obtain crystallization and melting parameters.

### 2.3. Preparation of Edible Films

Edible films were prepared by the casting technique. Corn starch solutions (1.5%, *w/v*) were obtained by dispersing Maizena powder in water at 60 °C for 30 min under stirring (100 rpm) to induce starch gelatinization [12]. Then, 0.5 wt% of glycerol and OE (0.05, 0.1 and 0.2 wt%) was added and heated at 60 °C for 10 min under stirring (100 rpm). A total of 30 mL of film-forming dispersions were poured into Petri dishes (18 cm diameter) and dried at 60 °C for 24 h in an oven. Then, they were peeled off the plates and conditioned at 50% relative humidity (RH) and 23 ± 1 °C in a climate chamber (Dycometal, Barcelona, Spain) before analyses. The obtained edible films were named as controls, OE 0.05, OE 0.1 and OE 0.2, where the number corresponds to the OE content (wt%).

### 2.4. Characterization of Edible Films

#### 2.4.1. Thickness and Color Measurement

The thickness of films was measured, in triplicate, with a precision of 0.001 mm, by using a digital micrometer (293 MDC-Lite Digimatic Micrometer, Mitutoyo, Japan) at five random positions, after 48 h of conditioning at 50% RH and 23 ± 1 °C.

Color is a key factor to be considered in food packaging films since it may be decisive from an aesthetic point of view and the consumer acceptance of the food product. Color properties of films were studied, in triplicate, using a spectrophotometer Konica CM-3600d (Konica Minolta Sensing Europe, Valencia, Spain) using the CIELAB color notation system (International Commission on Illumination). Three color properties were studied: lightness (L*-axis), which goes from 0 or absolute black to 100 or perfect white; saturation (a*-axis), which goes from positive axis with red color to negative ones with green shares;, finally, the tone angle (b*-axis), which goes from a positive yellow axis to negative blue ones. The measured coordinates were used to calculate the total color difference (ΔE) and whiteness index (WI), with respect to the control film, as given by Equations (2) and (3) [31].

∆E = [(∆L*)^2^ + (∆a*)^2^ + (∆b*)^2^]^1/2^ (2)

WI = 100 − [(100 − L*)^2^ +a*^2^ +b*^2^] ^½^ (3)

#### 2.4.2. Structural Characterization

The structural characterization of films (1.5 × 1.5 cm^2^) was carried out by ATR-FTIR as it was described in Section 2.2.5. Three measurements were obtained for each sample.

#### 2.4.3. Thermal Characterization

TGA and DSC tests were carried out using the same instruments and conditions previously described in Section 2.2.6.

The effect of the OE incorporation on the oxidative stability of films was studied by means of the oxidation onset temperature (OOT) by DSC [51]. Film samples (sample weight of 8.0 ± 0.1 mg) were heated up to 300 °C at 10 °C min^−1^ under oxygen atmosphere (gas flow rate at 50 mL min^−1^). The OOT value can be determined from the onset temperature of the exotherm observed in the temperature scanning experiment.

#### 2.4.4. Mechanical Properties

Three tensile parameters were obtained from the stress–strain curves as indicated in the ASTM D882-09 standard [52]: the elastic modulus, defined as the slope of the stress–strain curve in the elastic deformation region that measures the sample resistance to being deformed elastically; the tensile strength, defined as the point of maximum stress that the material is capable of withstanding without fracture; the elongation at break as the point at which the sample finally breaks. Tensile tests were carried out with a 3340 Series Single Column System Instron Instrument, LR30K model (Lloyd Instruments Ltd, Fareham Hants, UK) equipped with a 2 kN load cell. Tests were performed at room temperature in rectangular probes (100 × 10 mm^2^), using an initial grip separation of 50 mm and a crosshead speed of 5 mm min^−1^. Five replicates of each sample, previously conditioned at 23 ± 1 °C and 50% RH, were performed and mean values were reported.

Shore D hardness of films was determined with a JBA 673-D durometer (Instruments J. Bolt S.A., Barcelona, Spain), following the UNE-EN ISO 868:2003 standard [53]. Five specimens were tested for the different tests.

#### 2.4.5. Barrier Properties

The oxygen transmission rate (OTR) was determined by following the procedure already described by Valdés et al. [49] with an oxygen permeation analyzer (8500 model Systech Instruments, Metrotec S.A., Spain). Results were expressed as oxygen transmission rate per film thickness (OTR*e). Tests were performed in triplicate.

The solubility of films was determined, in triplicate, as previously detailed [54]. Pieces of each film (2 × 2 cm^2^) were dried in an oven at 60 ± 1 °C for 24 h. The moisture content was determined, in triplicate, by the difference resulting from the initial and final weights. Then, to study the film solubility, samples were introduced in 50 mL of distilled water for 10 min at 30 ± 1 °C under constant stirring. Films were dried to constant weight and losses of soluble material were determined by weight difference and expressed as the percentage of humidity [55].

Water vapor permeability (WVP) determinations were performed by using CaCl_2_ at 23 ± 1 °C and 50% RH in a climate chamber (Dycometal, Barcelona, Spain) as indicated in the UNE 53097:2002 standard [56]. Three measurements were obtained for each sample.

### 2.5. Antioxidant Activity of Edible Films

The evaluation of the antioxidant capacity of the active edible films with OE addition was performed using the method reported by Hafsa et al. [57] with a slight modification (ethanol was used instead of methanol during extraction). Film samples, sized 1.5 × 1.5 cm^2^, were placed in tubes with 5 mL of 96 wt% ethanol at 25 ± 1 °C under stirring (100 rpm) in the dark for 24 h. Then, antioxidant assays (DPPH, ABTS and FRAP) were performed as it was previously described in Section 2.2.3 using the ethanolic fraction obtained after film extraction as the sample. Analyses were carried out in triplicate.

### 2.6. Antimicrobial Activity of Edible Films

The antimicrobial activity of the developed active edible films was investigated against Gram-negative (*E. coli*, CECT 434) and Gram-positive (*S. aureus*, CECT 239) bacteria. The optical density method was followed by using the Biomate-3 UV-Vis spectrophotometer at 540 nm [58]. In this method, the turbidity measurement can be used as an indicator of the bacterial growth since the transmission of light decreases as the cell population increases. The spectrophotometric measurements were performed after the incubation of edible films (1.0 cm width × 4.5 cm length) into 10 mL of each bacterial suspension adjusted to the McFarland standard of approximately 10^5^ CFU mL^−1^ in saline solution at 37 ± 1 °C for 18 h. Bacterial growth was quantified and compared to blanks in the absence of films. Triplicates were prepared for each sample.

### 2.7. Statistical Analysis

ANOVA and Tukey’s tests were applied to all the obtained results with the SPSS software (Version 15.0, Chicago, IL, USA) at a confidence level of 95% (*p* < 0.05).

## 3. Results and Discussion

### 3.1. Olive Extract Characterization

#### 3.1.1. Antioxidant Activity, TPC and TFC

The antioxidant activity of OE was evaluated by using three different spectrophotometric methods: two single electron transfer (SET) assays (DPPH and FRAP) and one hydrogen atom transfer (HAT) assay (ABTS) [59]. Similar results were obtained for OE (100 mg kg^−1^ ethanol) in the three assays performed with a percentage of inhibition values of 35.92 ± 0.01%, 26.41 ± 0.01% and 33.18 ± 0.01% for DPPH, FRAP and ABTS, respectively. These results revealed the high antioxidant activity of OE by all tested methods. Similar results were reported in the literature for oils extracted from Empeltre and Arbequina olive cultivars [35].

Phenolic compounds derived from plant residues have raised some attention due to their functional and nutritional benefits, such as their antioxidant and antimicrobial activity. TPC was also determined [2,3,60] in OE with a value of 71.86 ± 0.01 mg GAE g^−1^. The high antioxidant activity of OE can be attributed to the presence of active compounds such as oleuropein and ligstroside, which are the main active compounds present in green olive fruits, leaves and table olives. Downstream metabolites such as oleacein, oleocanthal, hydroxytyrosil-elenolate, tyrosil-elenolate, oleoside-11-methyl ester, elenoic acid, hydroxytyrosol, and tyrosol have also shown antioxidant activity [37,38]. According to Sivakumar et al. [61], metabolites of oleuropein and ligstroside are considered olive bioactives which exert antioxidants via free radical processes and electrophilic molecular dynamics. Among the phenolic compounds reported in the literature from different olive oils [60,61], hydroxytyrosol, tyrosol, naringenin, caffeic and cinnamic acids showed a strong positive correlation (*r* > 0.90) with the antioxidant capacity of the studied oils. Some authors stated that the antioxidant effect of OE is mainly due to hydroxytyrosol, oleuropein and tyrosol [62]. In the case of oleuropein, the antioxidant effect was primarily attributed to the donating electron ability mainly due to the presence of hydroxyl groups (particularly the 1, 2-Dihydroxybenzene moiety) in its chemical structure. These hydroxyl groups could donate hydrogen to prevent oxidation [37]. According to this information, phenolic compounds present in the OE may be responsible of the powerful antioxidant capacity of OE extracted from Spanish olive fruit by-products, in particular by hydroxytyrosol which is present in OE in a concentration of nearly 41 wt%.

Regarding flavonoids, OE showed a value of 0.873 ± 0.008 mg CAE g^−1^ which was higher than the values reported by different authors in olive oils [35]. According to them, the major flavonoids quantified in different olive oils were rutin, quercetin, luteolin, naringenin, catechin and apigenin. Thus, the findings obtained in our study have demonstrated that the studied OE shows noticeable free radical scavenging properties, which can be related to its high contents of phenolic compounds and, among them, flavonoids.

#### 3.1.2. Antimicrobial Activity

**Figure 1:**
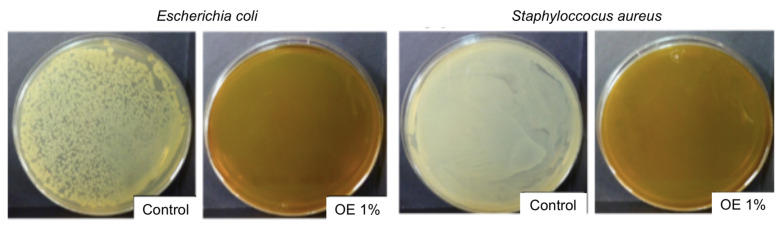
Antimicrobial activity of olive extract (OE) at 1 wt% observed by using the agar-plate diffusion method.

As it has been discussed in the previous section, OE revealed a high TPC value which was in agreement with those values obtained for different monovarietal olive oils [59,61]. No bacterial growth was observed for OE at 1 and 5 wt% in contrast to the positive controls, as it is shown in Figure 1 for OE at 1 wt%, as an example. These results suggested the high antimicrobial effect of OE against *E.coli* and *S.aureus* bacterial strains and they are in agreement with the antibacterial activity of polyphenols reported in the literature for olive oil [60].

Moreover, Janakat et al. [48] reported the potent antimicrobial activity of *amurca* (olive oil lees) extracts against different Gram-positive, Gram-negative and foodborne pathogens and this effect was related to the high content of phenolic compounds present in the amurca extract.

#### 3.1.3. ATR-FTIR

**Figure 2:**
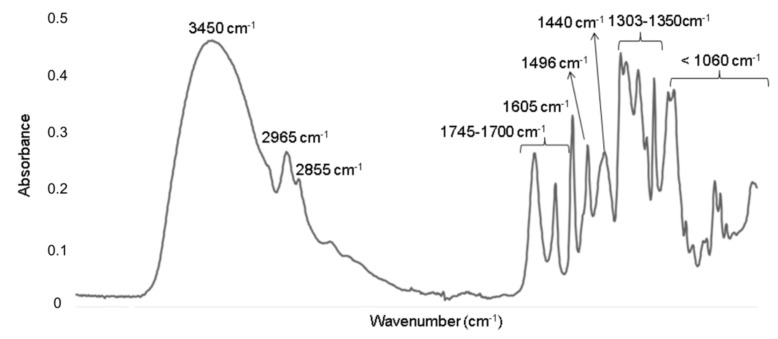
FTIR spectrum obtained for OE.

The FTIR spectrum of OE revealed the typical characteristic absorption bands associated to common oils (Figure 2) [14,63,64,65] at the following wavenumbers: 3450 cm^−1^, which was associated to the OH stretching vibration mode of polyphenols, mono- or diglycerides or water; 2965 cm^−1^ (asymmetric stretching vibration of C-H in aliphatic CH_3_ groups) corresponding to the alkyl rest of triglycerides mostly present in vegetable oils; 2855 cm^−1^ (symmetric stretching vibration of C-H of aliphatic CH_2_ group); 1745 and 1712 cm^−1^ (stretching vibration of the ester carbonyl functional groups in triglycerides and free fatty acids, C = O), 1605 cm^−1^ (C = C stretching vibration of cis olefins, HC = CH), 1496 and 1440 cm^−1^ (bending vibration of C-H of CH_2_ and CH_3_ aliphatic groups, respectively), 1303–1350 cm^−1^ (bending in plane vibrations of C-H bonds of cis-olefinic groups), 1096 cm^−1^ (stretching vibration of C-O ester groups), 967 cm^−1^ (bending vibration of CH functional groups of isolated trans-olefin), 914 cm^−1^ (bending vibration of cis -HC = CH- group) and 722 cm^−1^ (overlapping of CH_2_ rocking vibration and the out-of-plane vibration of the cis -HC = CH− group of disubstituted olefins).

Polyphenols, such as cinnamic acids, are characterized by the presence of the CH_2_ = CH-COOH group in their structure, contributing to the high antioxidant capacity of OE [35], although it is possible that the overall antioxidant potential of the studied extract could be due to the combined effect of phenolic acids and other antioxidant components present in olive samples [35].

#### 3.1.4. Thermal Characterization

**Figure 3:**
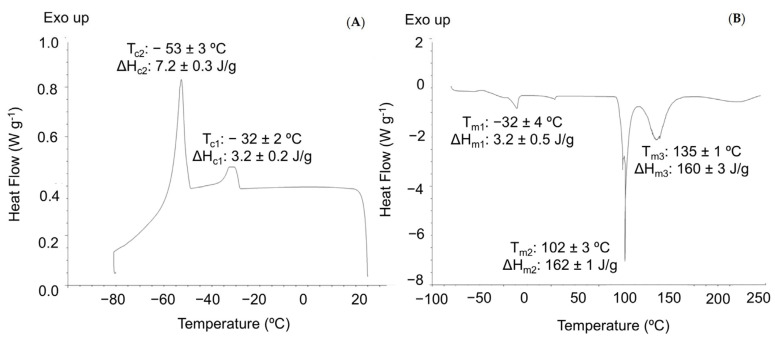
Differential scanning calorimetry (DSC) curves and parameters obtained for crystallization (**A**) and melting (**B**) transitions.

The thermal characterization of OE was studied by DSC and TGA. Figure 3 shows the DSC thermograms obtained which gave valuable information on the thermal properties of the sample. The cooling profile obtained for OE (Figure 3A) was similar to that previously reported in the literature for some vegetable oils such as olive pomace acid oil [64] and canola oil [66]. Two main exothermic transitions related to the crystallization of triunsaturated triacylglycerols (TAGs) at −53 °C and a minor exothermic event at around −32 °C related to the crystallization of desaturated TAGs were observed. A comparable cooling profile was reported in vegetable oils with high oleic acid and unsaturated fatty acids, such as linoleic acid, content [67].

The DSC heating thermograms of vegetable oils normally show a larger number of thermal events than cooling ones, by the overlapping of melting events in several polymorphic forms of crystals. OE showed three main endothermic events at around −32, 102 and 135 °C that may be ascribed to the melting of TAGs with a combination of unsaturated and saturated fatty acids [67] (Figure 3B).

**Figure 4:**
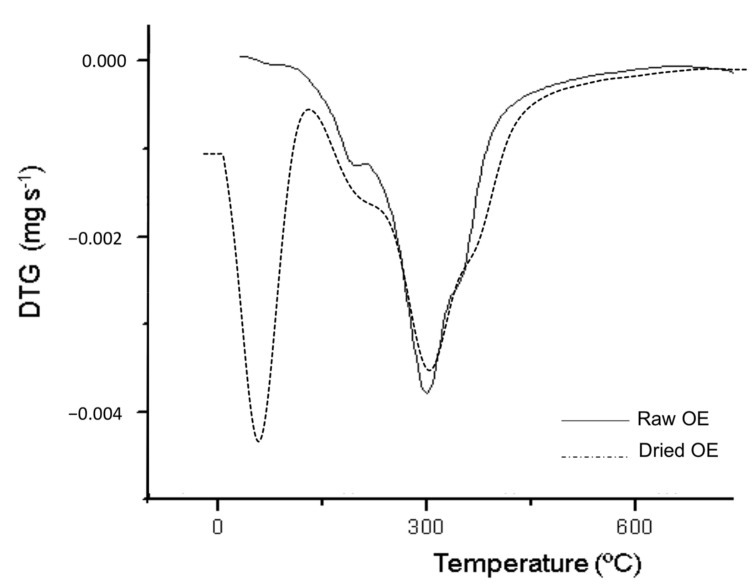
Derivative thermogravimetric (DTG) curves obtained for raw and dried OE.

Derivative thermogravimetric (DTG) curves obtained for raw and dried OE are shown in Figure 4. Raw OE showed a high content of water and volatile compounds at around 100 °C in agreement with the melting transition observed in DSC at 102 ± 3 °C. The dried OE showed three main decomposition phases, at 224 ± 2 °C, 299 ± 2 °C and 359 ± 1 °C, which were related to the degradation of polyunsaturated (PUFAs), monounsaturated (MUFAs), mainly oleic acid, and saturated (SFAs) fatty acids, respectively [68]. Finally, T_ini_ was obtained at 189 ± 3 °C, concluding that film processing will not affect the thermal stability of the antioxidant compounds present in the studied active extract.

### 3.2. Characterization of Edible Films

#### 3.2.1. Visual Appearance, Thickness and Color

**Figure 5:**
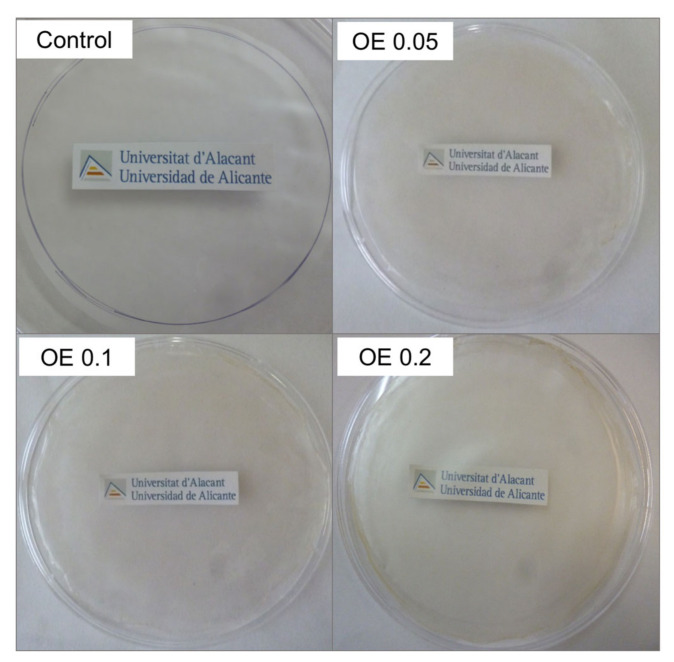
Visual appearance of the obtained edible films.

The visual appearance of the obtained films is shown in Figure 5. In general, as OE content increased a lower transparency of films was observed compared to the control film, showing active films of a slight yellow color. A significant (*p* < 0.05) slight decrease in L* (lightness) values and increase in a* and b* parameters with increasing OE addition (Table 1) was obtained. Concerning the total color difference (ΔE), a clearly significant (*p* < 0.05) increase was observed with OE concentration, being higher for films added with 0.02 wt% OE. However, WI values followed a decreasing tendency with increasing OE loading. It should be highlighted that the chromaticity parameters of the color CIELab space were obtained on a standard white plate, so the decrease in WI and L* values with respect to the control film may reflect a lower transparency. In this sense, the incorporation of OE contributes to intensify the color of active films [31] due to the natural yellowish color of the OE.

No significant differences between films were observed regarding thickness with an average value around 0.09 mm. Thickness is an important issue to be considered as changes in structural, mechanical, thermal or barrier properties should be expected in films presenting nonhomogeneous thickness values. Similar thickness and color parameters were obtained for edible chitosan-based films added with extra virgin olive oil emulsion films at concentrations ranging from 5 to 15 wt% [69] and chitosan/glycerol/olive oil (0.2 wt%) films containing dispersed cellulose nanocrystals [31].

#### 3.2.2. Structural Characterization by ATR-FTIR

**Figure 6:**
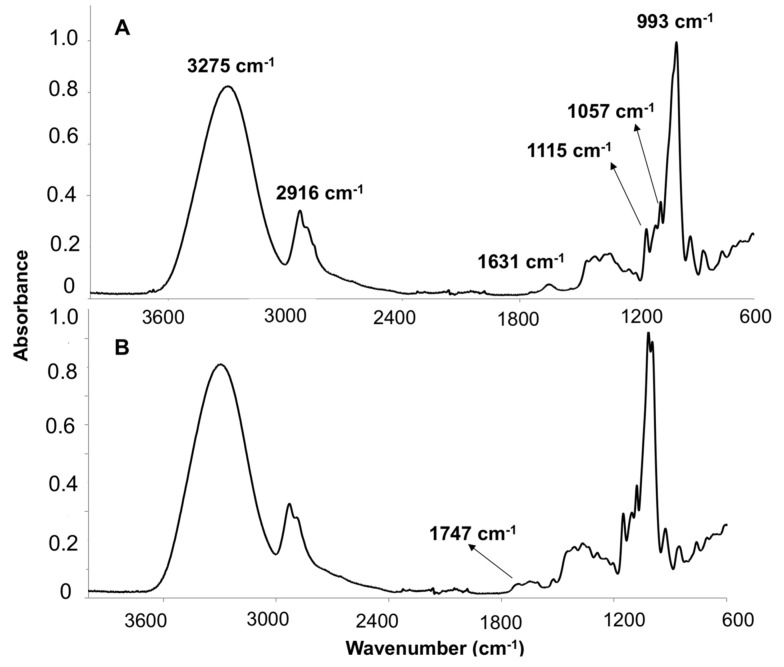
FTIR spectra of control film (**A**) and active film with 0.2 wt% of OE (**B**).

The FTIR analysis was performed to study the effect of OE addition into the corn starch matrix and the results obtained are shown in Figure 6. The structural characterization of the edible films showed the characteristic bands attributed to the main components of starch [70]. Although no experimental analysis was carried out to determine the chemical composition of the corn starch matrix used in this work, the nutritional composition reported by the supplier indicated that Maizena is composed of 86 wt% of carbohydrates, 1.0 wt% dietetic fiber, 0.4 wt% proteins and less than 0.5 wt% fats [71]. Among carbohydrates, amylose (25–35 g 100 g^−1^ of starch) containing only α-1, 4-glycoside linkages and amylopectin (65–75 g 100 g^−1^ of starch) containing a 20:1 α-1, 4 to α-1, 6 linkage ratio [72,73] are responsible for the film-forming capacity of this polymer matrix.

The spectrum obtained for the control film (Figure 6A) showed the characteristic bands of C-O-H present in starch around 1115 and 1057 cm^−1^, while the band at 993 cm^−1^ was characteristic of the anhydroglucose ring O-C stretching. Water adsorbed in the starch amorphous region can be identified as the broad infrared band located at 1631 cm^−1^. The overlapped bands around 2916 cm^−1^ were attributed to the asymmetric and symmetric stretching vibrations of −CH bonds in starch CH_2_ groups. Finally, a very broad intense band due to hydrogen-bonded hydroxyl groups appeared at 3275 cm^−1^. This band was attributed to the complex stretching vibrations associated to free, inter- and intramolecular-bound hydroxyl groups which are abundant in starch, glycerol, and water (both free and adsorbed) molecules [15] The ATR-FTIR spectra of films added with OE showed similar infrared profiles, observing an additional band at 1747 cm^−1^ for films containing 0.2 wt% of OE (Figure 6B) which was ascribed to carboxyl and ester carbonyl groups. The presence of this band suggests that chemical linkages between the starch/glycerol matrix and the OE could occur since the FTIR spectra of OE (Figure 2) showed a stretching vibration corresponding to the ester carbonyl functional groups of triglycerides and free fatty acids [14,63,64,65]. Similar results were reported for flour-based films added with sorbitol (plasticizer) [69] and starch-based films loaded with lemongrass essential oil [15].

#### 3.2.3. Thermal Characterization

DSC results showed a good miscibility between all compounds in the film-forming process since only one melting transition phase was observed for all films around 210–220 °C (Table 2) with a melting enthalpy around 65–75 J g^−1^. A DSC endotherm transition around 230 °C for corn starch has been reported in agreement with the results obtained in this work [74]. Similar results were also reported for nano-SiO_2_/starch/PVA biodegradable films prepared by coating [75].

The incorporation of a plasticizer is usually needed to improve the processability of the polymer films by increasing their internal lubrication. In this study, glycerol (0.5 wt%) was used to reduce intermolecular forces and to increase the mobility of the polymer chains. The calorimetric curves obtained for all films showed a T_g_ around −60 °C which was related to glycerol. Similar results were reported by Arık Kibar et al., who observed a glass transition temperature of glycerol around −70 and −60 °C for corn starch–carboxymethylcellulose/methylcellulose biodegradable films [76]. No significant differences (*p* > 0.05) were observed regarding DSC parameters (Table 2) for the obtained films at the studied OE concentrations.

**Figure 7:**
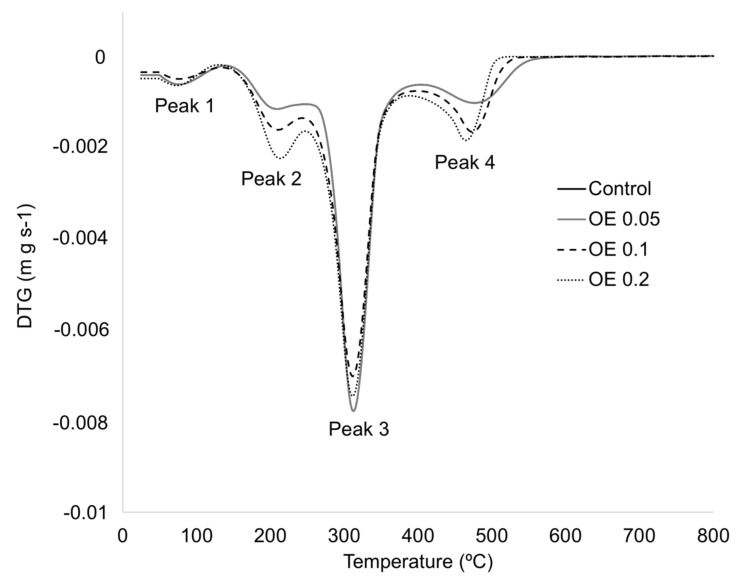
Derivative thermogravimetric (DTG) curves obtained for edible films.

The DTG curves obtained by TGA (Figure 7) showed two degradation peaks between 80 and 200 °C which were related to the elimination of bound water (peak 1) and the evaporation of glycerol and OE (peak 2) [73,77]. The third observed peak around 310 °C corresponded to the starch degradation with a final decomposition stage (peak 4) around 480 °C which was associated to the thermal decomposition and carbonization of the polymer matrix and OE. According to Liu et al. [74], three stages were identified for the thermal decomposition mechanism of starch. The first stage corresponds to the physical dehydration around 100 °C, followed by a second step associated to chemical dehydration and thermal decomposition. The main degradation peak starting at around 300 °C can be attributed to the thermal condensation reaction between hydroxyl groups of different starch chains and the evolution of water and other small molecular species, such as aldehydes [15]. When the sample was heated up to 500 °C, some carbonization processes were observed. In general terms, no significant differences (*p* > 0.05) between thermal parameters were observed between the different tested film samples (Table 2).

The obtained edible films added with OE significantly improved (*p* < 0.05) their thermo-oxidative stability compared to the control film according to the values obtained for the thermal parameters included in Table 3. As it can be seen, T_ini_ and OOT tend to increase with increasing OE content with values of 123 ± 3 °C and 102 ± 7 °C for the control film and 175 ± 1 °C and 152 ± 1 °C for OE 0.2, respectively. This behavior suggests that the addition of the olive extract allows the thermo-oxidative stability of the polymer matrix to increase, being noticeable for the active film added with 0.2 wt% OE. These results are in accordance with the DPPH, ABTS and FRAP values obtained for OE and they could be related to some antioxidant activity of phenolic compounds present in the active films, in agreement with the TPC and flavonoids results shown for OE.

#### 3.2.4. Mechanical and Barrier Properties

Mechanical and barrier properties (Table 3) were studied in the developed edible films. Regarding mechanical properties, control films showed a Young’s modulus of 3.4 ± 0.1 MPa, elongation at break of 67 ± 3% and shore D hardness of 76.21 ± 0.01. The incorporation of OE into the starch/glycerol matrix resulted in a significant decrease (*p* < 0.05) in mechanical parameters compared to the values obtained for the control film, in particular for the OE 0.2 film. Similar results were reported for composite films based on corn starch containing 0–0.5 wt% cassia oil [78]. Thus, it could be supposed that glycerol could interact with starch and the OE, respectively, forming micelles with OE in the film-forming liquid. The interaction of hydrogen bonds between starch and glycerol–OE micelles could replace the interaction of internal hydrogen bonds, decreasing the films mechanical properties and increasing the fluidity in the obtained films added with OE, being noticeable at 0.2 wt % OE loading. These results are in agreement with those obtained by FTIR in films with 0.2 wt% of the olive extract compared to the control film (Figure 6).

Regarding barrier properties, no significant differences (*p* > 0.05) were observed for moisture content and OTR values between samples. However, as it can be seen in Table 3, the addition of OE resulted in a significant (*p* < 0.05) effect on the WVP of the starch–glycerol films. In this sense, an increase in OE content resulted in a downward trend in WVP values, with the control film showing the highest value and presenting the minimum value (15 ± 2 kg m Pa^−1^ s^−1^ m^−2^) at the highest OE loading (0.2 wt%). The addition of some oils such as cassia oil [79] and olive oil [31] into polysaccharide matrices has been reported to decrease WVP values by the addition of hydrophobic lipids to hydrophilic polymer films, improving water resistance [16]. The interaction between all components in the film samples increased their density, thus reducing their water vapor transmission rate. The oil addition promotes the formation of emulsions within droplets stabilized by the colloidal particles distributed within the starch matrix increasing their stability, providing hydrophobic character and reducing the adsorption of water molecules [31,74]. As a result, OE addition increased the number of possibilities of rearrangement for starch chains and glycerol by their higher mobility, decreasing the number of hydroxyl groups available for the polysaccharide-water interactions, and finally resulting in the formation of hydrogen bonds between glycerol/OE and the hydroxyl groups of starch [15,80].

### 3.3. Antioxidant Activity

The antioxidant activity of the active edible films was studied by DPPH, ABTS and FRAP methods using the ethanolic fraction obtained after film extraction as a sample [57]. As it was expected, control films did not show an appreciable antioxidant activity (Table 4). However, the scavenging capacities of starch-based films incorporated with OE significantly increased (*p* < 0.05) with the OE concentration. Thus, as OE content increased higher DPPH, ABTS and FRAP values were obtained, which may be linked with the antioxidant activity of the extract and the presence of bioactive compounds in the edible films, such as phenolic acids, from OE. In fact, the scavenging activities of the active films were dependent on OE concentration with results varying from 0.71 ± 0.25% to 96.02 ± 0.53% for DPPH, 0.69 ± 0.17% to 99.61 ± 0.11% for ABTS and 0.07 ± 1.38% to 89.38 ± 0.09% for FRAP, in the control film and films with 0.2 wt% OE, respectively. Some studies have evaluated the antioxidant activity of chitosan-based edible films with Thymus moroderi or Thymus piperella essential oils [80] and Eucalyptus globulus essential oil [57], showing that the antioxidant activity was dependent on the concentration of these essential oils.

### 3.4. Antimicrobial Activity

The results obtained from the antimicrobial tests performed in the active edible films by the optical density method are shown in Table 5. As it can be seen, the incorporation of OE conferred some antimicrobial power to the active films compared to the corresponding controls. In this sense, as the OE content increased, the microorganism growth significantly decreased, being more noticeable (*p* < 0.05) for S. aureus in the film containing 0.2 wt% OE, whereas, a slightly significant decrease (*p* < 0.05) was observed for E.coli in the same formulation. These results were related to the release of antimicrobial phenolic compounds present in OE from the edible film matrix during the antimicrobial test. Similar results were reported from cassava starch films and cinnamon essential oil release profiles monitored for 2 h [16].

## 4. Conclusions

The present work suggested the potential of corn starch-based edible films incorporated with a natural olive extract OE as sustainable food packaging systems with antioxidant/antimicrobial properties to prevent the oxidative deterioration of packaged foodstuff. The results of this study showed that the incorporation of OE at 0.05, 0.1 and 0.2 wt.% into starch/glycerol films induced high antioxidant and antimicrobial activity against *S.aureus* and *E.coli* bacteria to the resulting films, being noticeable for films with 0.2 wt% OE. This fact was positively related to the total phenolic content of the extract rich in antioxidants with redox properties, in particular caused by hydroxytyrosol, which is present in OE at a concentration of nearly 41 wt%. The interactions observed by FTIR between corn starch and OE in the presence of glycerol resulted in an improvement in water vapor permeability with the OE addition, which was associated to the hydrophobic character of the OE lipid fraction. Homogeneity was obtained for all films with high visual transparency, being a positive and desirable characteristic for food packaging. The thermo-oxidative resistance of the active edible films with temperature was also significantly improved compared with the control film, increasing OOT and T_ini_ values as the OE content increased. This study has proved the suitability of using commercial Maizena^®^ as a low-cost corn starch source to be used for the successful development of edible films to prevent the oxidative deterioration of packaged foodstuff such as nuts, seeds and sausages, with prices around EUR 3.38 per kg in contrast to commercial corn starch suppliers with average prices of EUR 29 per kg. The approach applied in this work also allows reducing the use of synthetic additives and generation of food wastes, contributing to a circular economy.

## Figures and Tables

**Table 1:** Thickness (*n* = 5) and color (*n* = 3) parameters obtained for edible films. Mean ± SD.

Parameter	Control	OE 0.05	OE 0.1	OE 0.2
Thickness (mm)	0.09 ± 0.01 ^a^	0.09 ± 0.02 ^a^	0.10 ± 0.01 ^a^	0.11 ± 0.01 ^a^
L*	64.58 ± 0.01 ^a^	60.27 ± 0.01 ^b^	62.09 ± 0.01 ^c^	57.59 ± 0.02 ^d^
a*	−1.27 ± 0.04 ^a^	−0.62 ± 0.02 ^b^	1.17 ± 0.02 ^c^	3.26 ± 0.04 ^d^
b*	5.41 ± 0.03 ^a^	10.33 ± 0.04 ^b^	15.66 ± 0.02 ^c^	18.76 ± 0.01 ^d^
ΔE	−3.63 ± 0.09 ^a^	−0.99 ± 0.02 ^b^	1.20 ± 0.02 ^c^	3.01 ± 0.04 ^d^
WI	64.15 ± 0.01 ^a^	58.95 ± 0.01 ^b^	58.97 ± 0.01 ^b^	53.52 ± 0.02 ^c^

Different superscripts (^a, b, c, d^) within the same row and parameter indicate statistically significant different values (*p* < 0.05); OE indicates olive extract.

**Table 2:** Thermal parameters obtained by DSC and thermogravimetric analysis (TGA) for edible films. Mean ± SD (*n* = 3).

Formulation	ΔH_m_ (J g^−1^)	T_m_ (°C)	T_g_ (°C)	T_Peak 1_ (°C)	T_Peak 2_ (°C)	T _Peak 3_ (°C)	T _Peak 4_ (°C)
Control	68 ± 1 ^a^	214 ± 1 ^a^	−63 ± 2 ^a^	82 ± 6 ^a^	220 ± 2 ^a^	310 ± 6 ^a^	480 ± 2 ^a^
OE 0.05	69 ± 4 ^a^	220 ± 2 ^a^	−66 ± 2 ^a^	79 ± 4 ^a^	221 ± 2 ^a^	316 ± 3 ^a^	482 ± 2 ^a^
OE 0.1	66 ± 1 ^a^	220 ± 1 ^a^	−63 ± 2 ^a^	77 ± 5 ^b^	220 ± 1 ^a^	312 ± 1 ^a^	476 ± 5 ^a^
OE 0.2	68 ± 5 ^a^	214 ± 4 ^a^	−59 ± 5 ^a^	76 ± 4 ^c^	225 ± 3 ^a^	312 ± 1 ^a^	479 ± 3 ^a^

Different superscripts (^a, b, c^) within the same column indicate statistically significant different values (*p* < 0.05); ΔH_m_: melting enthalpy transition; T_m_: melting temperature; T_g_: glass transition temperature; T_peak_: maximum degradation temperature.

**Table 3:** Mechanical (*n* = 5) and barrier (*n* = 3) parameters obtained for edible films. Mean ± SD.

Property	Control	OE 0.05	OE 0.1	OE 0.2
Young’s modulus (MPa)	3.4 ± 0.1 ^a^	4.1 ± 0.1 ^b^	4.6 ± 1.0 ^b^	2.1 ± 0.2 ^c^
Elongation at break (%)	67 ± 3 ^a^	42 ± 1 ^b^	41± 7 ^b,c^	47± 2 ^d^
Shore D hardness	76.21 ± 0.01 ^a^	64.20 ± 0.02 ^b^	54.42 ± 0.03 ^c^	46.84 ± 0.01 ^d^
Moisture content (%)	7.3 ± 0.2 ^a^	7.3 ± 0.4 ^a^	6.4 ± 0.8 ^a^	6.9 ± 0.4 ^a^
OTR (cm^3^ mm m^−2^ day)	0.49 ± 0.02 ^a^	0.41 ± 0.07 ^a^	0.40 ± 0.08 ^a^	0.38 ± 0.09 ^a^
WVP × 10^−4^ (Kg m Pa^−1^ s^−1^ m^−2^)	23 ± 2 ^a^	33 ± 5 ^b^	24 ± 1 ^a^	15 ± 2 ^c^

Different superscripts (^a, b, c, d^) within the same raw and parameter indicate statistically significant different values (*p* < 0.05).

**Table 4:** Antioxidant activity and thermal parameters obtained for edible films. Mean ± SD (*n* = 3).

Formulation	DPPH (%)	ABTS (%)	FRAP (%)	T_ini5%_ (°C)	OOT (°C)
Control	0.71 ± 0.25 ^a^	0.69 ± 0.17 ^a^	0.07 ± 1.38 ^a^	102 ± 7 ^a^	123 ± 3 ^a^
OE 0.05	22.42 ± 0.17 ^b^	99.38 ± 0.22 ^b^	16.30 ± 3.62 ^b^	110 ± 6 ^a^	156 ± 3 ^b^
OE 0.1	94.00 ± 1.00 ^c^	99.67 ± 0.11 ^b^	74.25 ± 1.35 ^c^	137 ± 9 ^b^	178 ± 1 ^c^
OE 0.2	96.02 ± 0.53 ^d^	99.61 ± 0.11 ^b^	89.38 ± 0.09 ^d^	152 ± 1 ^c^	175 ± 1 ^c^

Different superscripts (^a, b, c^) within the same column indicate statistically significant different values (*p* < 0.05); DPPH indicates 1,1-dipheny l-2-picrylhydrazyl; ABTS indicates 2,2-azino-bis (3-ethylbenzothiazoline-6-sulfonic acid); FRAP indicates ferric reducing antioxidant power; T_ini_ indicates initial degradation temperature; OOT indicates oxidation onset temperature.

**Table 5:** Absorbance values obtained for antimicrobial activity (optical density method) of active edible films. Mean ± SD (*n* = 3).

Formulation	*E. coli*	*S. aureus*
Control	0.159 ± 0.009 ^a^	0.192 ± 0.004 ^a^
OE 0.05	0.153 ± 0.002 ^a^	0.166 ± 0.002 ^a,b^
OE 0.1	0.149 ± 0.008 ^a^	0.136 ± 0.019 ^b^
OE 0.2	0.143 ± 0.006 ^b^	0.105 ± 0.001 ^c^

Different superscripts (^a, b, c^) within the same column indicate statistically significant differences (*p* < 0.05).
